# Evaluation of six novel antigens as potential biomarkers for the early immunodiagnosis of schistosomiasis

**DOI:** 10.1186/s13071-015-1048-2

**Published:** 2015-09-04

**Authors:** Yuanbin Zhang, Jing Zhao, Xinye Wang, Xindong Xu, Weiqing Pan

**Affiliations:** Institute for Infectious Diseases and Vaccine Development, Tongji University School of Medicine, Shanghai, 200092 China; Department of Tropical Infectious Diseases, Second Military Medical University, Shanghai, 200433 China

**Keywords:** *Schistosoma japonicum*, Schistosomiasis, Biomarker, Early diagnosis, Animal model

## Abstract

**Background:**

Early diagnosis of schistosomiasis, prior to egg laying, would enable earlier treatment and help interrupt the transmission cycle of the parasite and the progress of the disease. Previously we identified six novel antigens with potential as diagnostic markers for human *Schistosoma japonicum* infections. In this study, we evaluated these antigens as candidate biomarkers for the early diagnosis of schistosomiasis in mice and rabbits.

**Methods:**

The transcriptional profiles of the six antigens (SjSP-13, SjSP-23, SjSP-160, SjSP-164, SjSP-189 and SjSP-216) at different developmental stages were analyzed by quantitative PCR. The recombinant proteins were expressed in *E. coli* and purified with nickel chelate affinity chromatography. We then developed recombinant protein-based ELISA kits to analyze the kinetics of antigen-specific antibodies during the course of infection in mice and rabbits. The early diagnostic validity of the candidate SjSP-216 was further evaluated in mice and rabbits infected with *S. japonicum*.

**Results:**

Of the six antigens, SjSP-13, SjSP-160 and SjSP-216 were highly expressed in 21-day old young worms, while SjSP-23, SjSP-164 and SjSP-189 were highly expressed in eggs. In the mouse model, we detected a significant increase in antibodies against SjSP-13 and SjSP-216 at 3 weeks post-infection. However, in the rabbit model, only anti-SjSP-216 antibody showed a significant increase at this time point. We recorded 100 % diagnostic sensitivity and specificity of SjSP-216-based ELISA in both infected mice and rabbits, 3 weeks after infection.

**Conclusions:**

This study strongly suggests that SjSP-216, a highly expressed gene in the young worms, could serve as a potential biomarker for the early immunodiagnosis of *S. japonicum* infections in vertebrate hosts.

**Electronic supplementary material:**

The online version of this article (doi:10.1186/s13071-015-1048-2) contains supplementary material, which is available to authorized users.

## Background

Schistosomiasis is a chronic disease caused by parasitic trematodes of the genus *Schistosoma* that afflicts over 200 million people worldwide and kills >300,000 people annually [1–3]. Humans are infected by cercariae, which are released from infected snails when they come in contact with contaminated water [4]. After the cercariae penetrate the skin, the parasites become schistosomula and over 4–6 weeks migrate and mature to adult male or female worms. Adult worms live as pairs in the portal and mesenteric veins (*S. japonicum* and *S. mansoni*) or in the veins of the bladder (*S. haematobium*). They are long-lived and produce hundreds of fertilized eggs per day [5]. Although most eggs are retained within the host tissues, about one-third are excreted into the environment and hatch into free-living miracidia, which can infect susceptible snails in water, thus completing the cycle [6, 7]. The host’s immune response to antigens excreted from the trapped eggs can induce severe morbidity, including hepatic fibrosis, portal hypertension, urinary obstruction and bladder carcinoma [8, 9].

One factor contributing to high disease prevalence and severe morbidity is the absence of effective diagnostic methods for detecting schistosome infections, especially in the early phase [10]. The traditional gold standard for the diagnosis of schistosomiasis involves detecting eggs in and hatching miracidium from the host’s feces or urine [11, 12]. However, in the majority of schistosome infections, the paired worms start to discharge eggs about 4 weeks after infection [13]. Thus, these diagnostic methods are relatively insensitive and cannot detect early infections. If schistosome infections could be detected prior to egg deposition, the source of infection would be controlled and subsequent treatment with Praziquantel^TM^ would effectively prevent the development of severe pathologic lesions.

Immunodiagnostic detection of antibodies against schistosome antigens is an attractive option for detecting early infections. However, currently available antibody detection assays are not suitable as they use antigens extracted from schistosome eggs or adult worms [14–16]. Previous studies have revealed that the reactivity of antibodies against crude worm antigens remains low until the infections become patent in the experimental hosts [17, 18]. Therefore, the ideal immunodiagnostic assay to detect early infections would use antigens from the schistosomula or cercariae [19]. However, it is notoriously difficult to extract antigens from the schistosomula or cercariae.

In a previous study, we used a high-throughput glutathione S-transferase (GST) fusion protein array assay to identify several antigens (SjSP-13, SjSP-23, SjSP-160, SjSP-164, SjSP-189 and SjSP-216) with the potential to be immunodiagnostic markers for human schistosomiasis [20]. In this study, we aim to verify the utility of these six antigens as candidates for the early diagnosis of *S. japonicum* infections in a murine and rabbit model.

## Methods

### Parasites and animals

A field-collected isolate of *S. japonicum* from Guichi County, Anhui Province, China was used in all the experiments. Parasites were maintained in *Oncomelania hupensis* snails and in rabbits. Female 12-week old New Zealand White rabbit and female 6–8 week old BALB/c mice were obtained from SLAC Laboratory Animal Co., Ltd. of the Chinese Academy of Sciences of Shanghai. All procedures performed on animals within this study were conducted in accordance with and by approval of the Internal Review Board of Tongji University School of Medicine.

### Real-time PCR

Young worms were recovered by perfusion from BALB/c mice that had been infected 3 weeks earlier with 200 cercariae. Adult worms were recovered by perfusion from mice 6 weeks post infection. Eggs were purified from livers of infected rabbits. Total RNAs were extracted from cercariae, young worms, adult worms and eggs using Trizol (Invitrogen, USA). First-strand cDNA was performed with the reverse transcriptase Superscript (Takara, Japan) with oligo (dT) primers using 1 μg total RNA as template. We then used real-time PCR to quantify gene expression levels. All real-time PCR were run in three replicates. Real-time quantification was performed using an Applied Biosystems 7300 Sequence Detection system using SYBR Premix Ex Taq Kit (Takara). Data were analyzed according to 2^−ΔΔCt^ method using GAPDH as the internal control for each sample. The fold-changes of gene transcriptional level in young worm, adult worm and egg were calculated relative to that of cercaria. The house keeping gene SOD was set as a control gene. All primers used for real-time PCR are listed in Table 1.

**Table 1:** Primers used for Real-Time PCR

Primer	Primer sequence (5′-3′)
SjSP-13 Forward	CTGTCGTTTACTGTGTGG
SjSP-13 Reverse	CCATTCTTCTTTTGGGAT
SjSP-23 Forward	AAGGCGGTATGATTCC
SjSP-23 Reverse	CCACGCACTCCTTGTTTTCTGA
SjSP-160 Forward	GGCGGGCATGGATTTAGTTC
SjSP-160 Reverse	GCTTGTAATGCCTTGC
SjSP-164 Forward	TTCACACACCCTTGGG
SjSP-164 Reverse	GTGATGGTGATGGTGATG
SjSP-189 Forward	TTAGGGTTCCGATTTAGTGC
SjSP-189 Reverse	CCGAGATAGGGTTGAGTGT
SjSP-216 Forward	GGATGCTGGATGGAAAGC
SjSP-216 Reverse	GAGGCCATTTCTTTCGTG
GAPDH Forward	GTGTTCCTACCCCCAATGTGT
GAPDH Reverse	GTCATACCAGGAAATGAGCTTGA
SOD Forward	CTGATGACGGAAAGGGAG
SOD Reverse	CTATGACACCACAAGCTACA

### Cloning, expression and purification of antigens

The gene fragments of antigens SjSP-13, SjSP-23, SjSP-160, SjSP-164, SjSP-189 and SjSP-216 (Genbank accession number: AY222880, AY814664, AY222887, AY814985, AY815838 and AY813624, respectively) were amplified from a mixture of cDNAs of cercariae, young worms, adult worms, and eggs by PCR with KOD polymerase (Toyobo, Japan). Specific primers with restriction enzyme sites are listed in Additional file 1. The PCR products were cloned into *E.coli* expression vector pGEX-4 T-1 (for SjSP-13, SjSP-160, SjSP-164, SjSP-189 and SjSP-216) or pET28a (for SjSP-23). The recombinant plasmids containing target DNA fragments were confirmed by DNA sequencing. Expression of recombinant proteins was induced with Isopropyl-D-1-thiogalactopyranoside (IPTG) at 1 mM. Recombinant proteins were purified from the insoluble inclusion body with a hexahistidine tag. The purified antigens were re-natured in refolding buffer C7 (1.0 mM TCEP, 250 mM NaCl, 12.5 mM β-cyclodextrin, 50 mM Tris–HCl pH 8.5) [20]. Protein concentration was determined by the Bradford method [21]. The predicted molecular weight of SjSP-23 and the GST fusion proteins of SjSP-13, SjSP-160, SjSP-164, SjSP-189, SjSP-216 were 13.0kD, 45.6kD, 51.9kD, 40.8kD, 41.1kD and 57.4kD, respectively.

### Indirect enzyme-linked immunosorbent assay

The 96-well microliter plates (Corning, USA) were coated with 100 μL per well of 1 to 2 μg/ml antigens diluted in coating buffer (0.05 M carbonate-bicarbonate, pH 9.6) for 16 h at 4 °C. The plates were washed 3 times with washing buffer (0.15 M phosphate buffer saline containing 0.05 % of Tween 20, pH 7.4). The free sites were saturated with 200 μL per well of blocking buffer (5 % skim milk dissolved in washing buffer) at 37 °C for 1 h. After washing three times, 100 μL of individual mouse sera (diluted 1:100) in blocking buffer were added to the plates and were incubated at 37 °C for 1 h. The plates were submitted to 5 times of washing and incubated at 37 °C for 1 h with goat anti-mouse IgG or goat anti-rabbit IgG conjugated with peroxidase (Abcam, USA) diluted in blocking buffer at the dilution of 1:20,000. Plates were washed again and 100 μL of TMB substrate solution was added to each well. The enzymatic reaction was stopped after 10 min of incubation at 37 °C by adding 50 μL per well of 2 N H_2_SO_4_. The results were obtained as absorbance values at 450 nm by a microplate reader.

### Serum collection

To analyze the dynamics of antigen specific antibodies during infection, three mice and three rabbits were infected with 30 ± 2 or 200 ± 10 cercariae by the subcutaneous route, respectively. Meanwhile, another three mice and three rabbits were used as non-infected controls. Serum samples were collected before infection and on week 1, 2, 3, 4, 5 and 6 after infection.

A separate experiment was performed to evaluate the early diagnostic validity of SjSP-216. Ten mice and six rabbits were infected the same as before; ten mice and six rabbits were used as a negative control of infection. Sera were collected at 3 weeks after infection.

### Statistic analysis

Data of real-time PCR were expressed as the mean ± standard deviation. The absorbance values were expressed as the mean ± standard error. All data were analyzed by the Student’s *t*-test, with a *p* value < 0.05 considered significant. We used Prism 4.0 software for all statistical analyses.

## Results

### Production of recombinant proteins

The gene sequences of the six proteins were verified as correct by DNA sequencing. The six recombinant proteins were successfully produced in *E.coli* (Additional file 2). The purified antigens were re-natured in refolding buffer C7 that was identified from the iFOLD Protein Refolding System 1 in our previous studies [20].

### Analysis of transcriptional profiles of the six antigens at different developmental stages

**Fig. 1:**
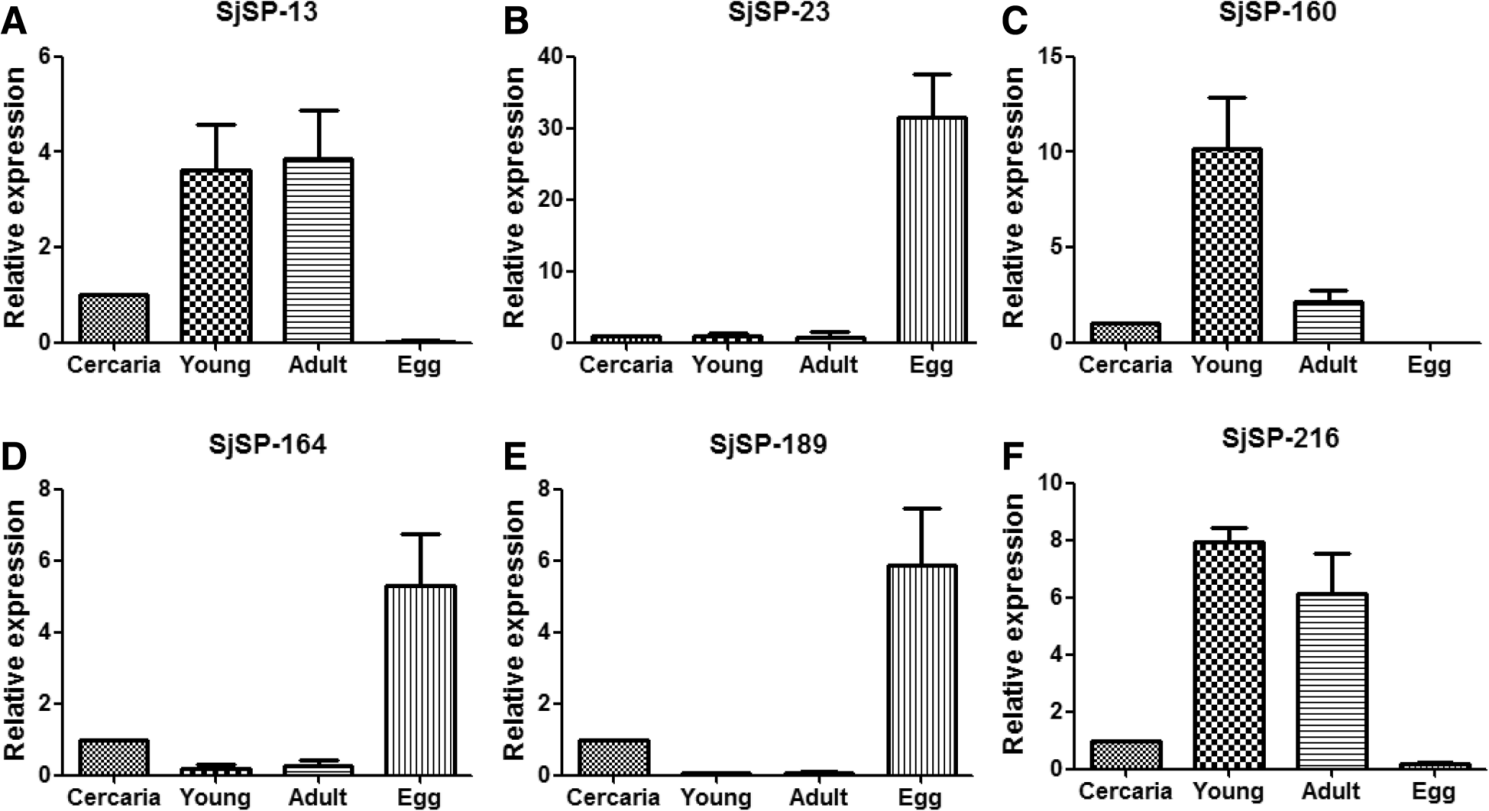
Differential expression profiles of the six novel antigens at different developmental stages of *S. japonicum*. Transcripts of the six antigens were detected by quantitative PCR at four life stages: cercaria, young worm (Young), adult worm (Adult) and egg. Data were analyzed according to 2^−ΔΔCt^ method using GAPDH as the internal control for each sample. The-fold changes of gene transcriptional level in young worm, adult worm and egg were calculated relative to that of the cercaria. Data were expressed as the mean ± standard deviation. **a** Expression profile of SjSP-13. **b** Expression profile of SjSP-23. **c** Expression profile of SjSP-160. **d** Expression profile of SjSP-164. **e** Expression profile of SjSP-189. **f** Expression profile of SjSP-216

The gene transcriptional profiles of the six antigens varied within the different developmental stages (Fig. 1). SjSP-13 (Fig. 1a), SjSP-160 (Fig. 1c) and SjSP-216 (Fig. 1f) were highly expressed in young worms and adult worms but poorly expressed in eggs. Conversely, the expression levels of SjSP-164 (Fig. 1d), SjSP-189 (Fig. 1e), and particularly SjSP-23 (Fig. 1b) were higher at the egg stage than the other stages. None of the six antigens were highly expressed in cercariae. As expected, the transcriptional level of the control gene, SOD, was similar at all developmental stages (Additional file 3).

### Kinetics of specific antibodies in the sera of mice infected with *S. japonicum*

**Fig. 2:**
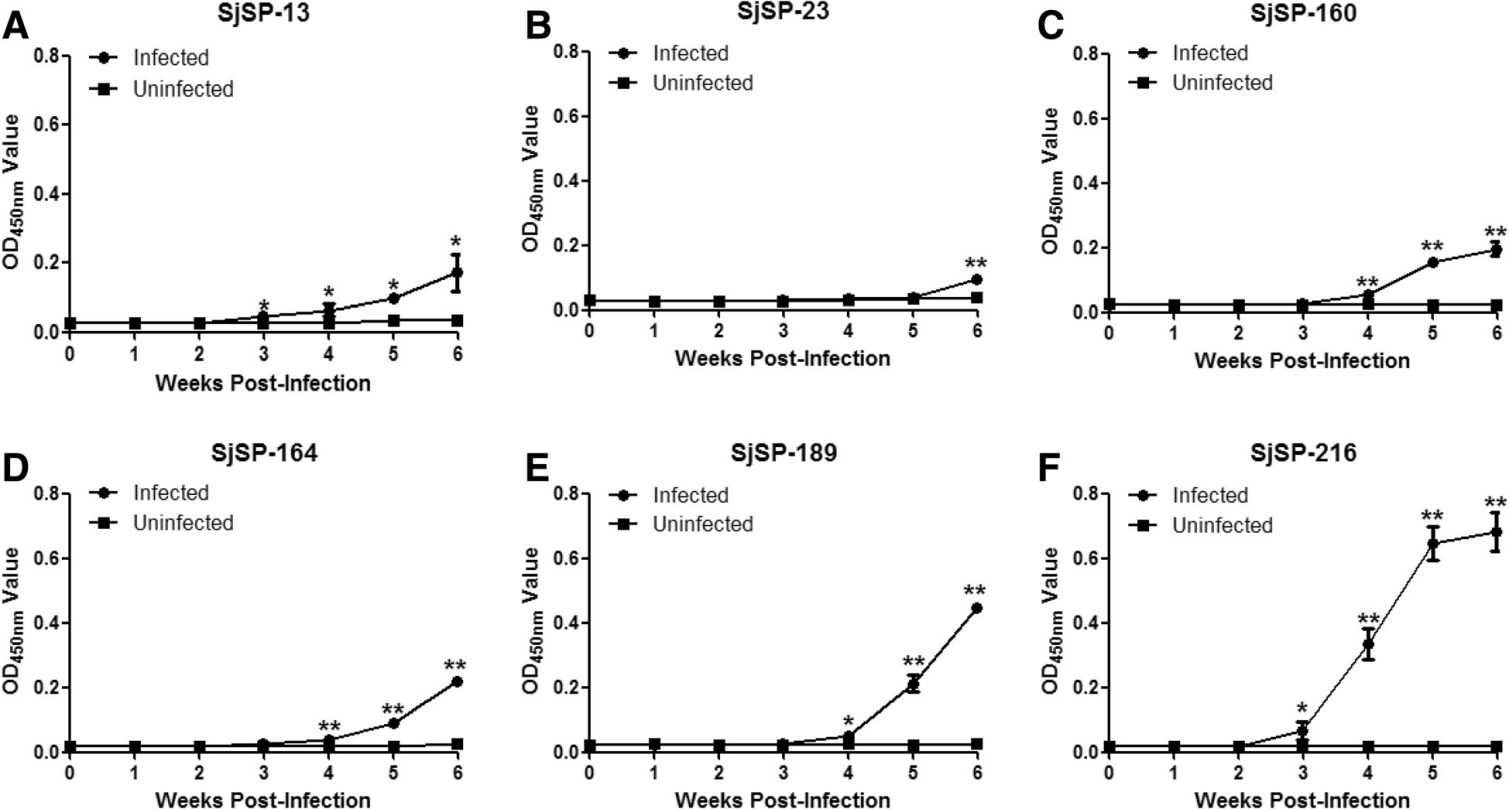
Kinetics of specific antibodies to the six antigens during the course of mouse schistosomiasis. Sera were collected from three infected and uninfected mice at different time points. The antigen-specific antibody was analyzed by ELISA. The absorbance values were expressed as the mean ± standard error. **a** Changes in anti-SjSP-13 antibody. **b** Changes in anti-SjSP-23 antibody. **c** Changes in anti-SjSP-160 antibody. **d** Changes in anti-SjSP-164 antibody. **e** Changes in anti-SjSP-189 antibody. **f** Changes in anti-SjSP-216 antibody

**Fig. 3:**
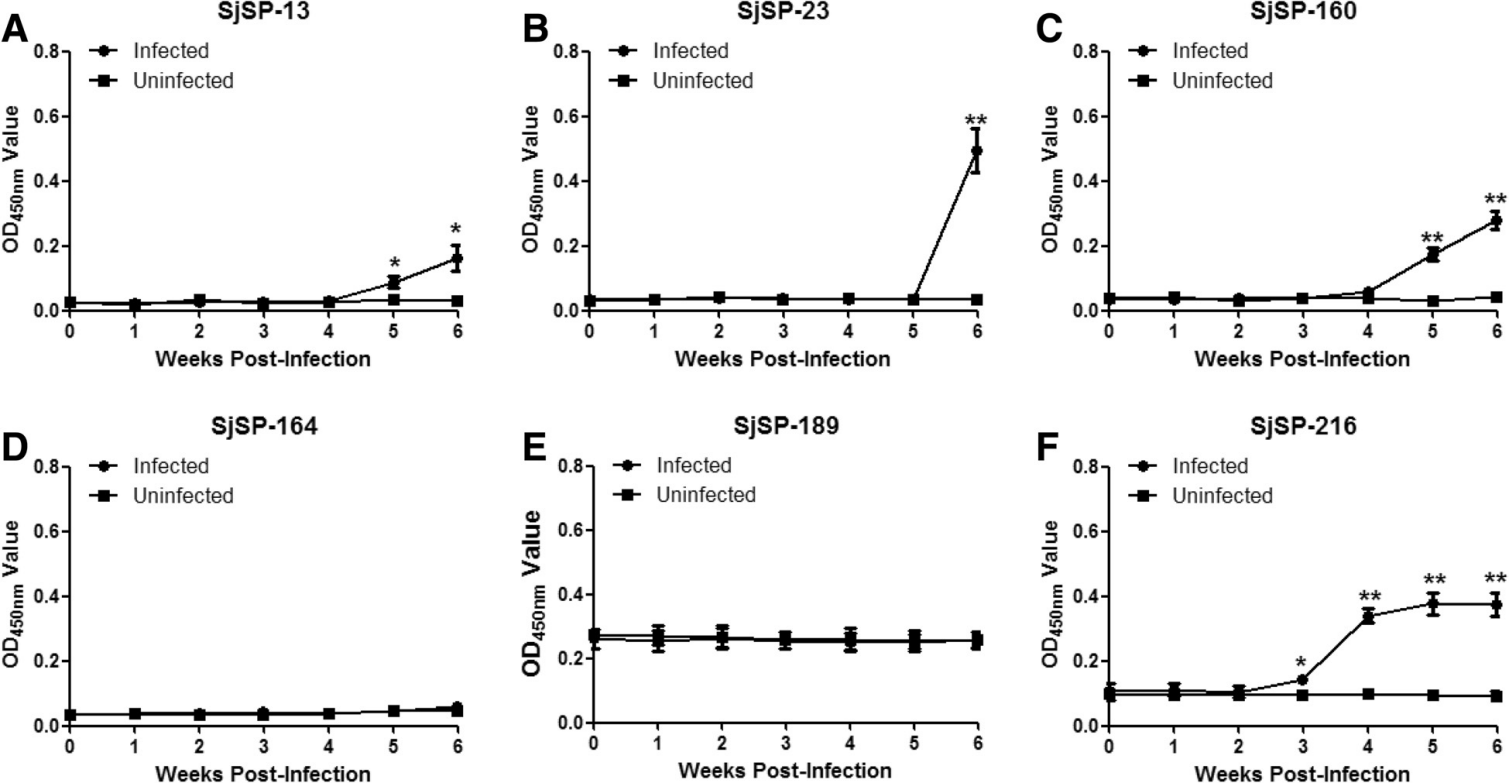
Kinetics of specific antibodies to the six antigens during the course of rabbit schistosomiasis. Sera were collected from three infected and uninfected rabbits at different time points. The antigen-specific antibody was analyzed by ELISA. The absorbance values were expressed as the mean ± standard error. **a** Changes in anti-SjSP-13 antibody. **b** Changes in anti-SjSP-23 antibody. **c** Changes in anti-SjSP-160 antibody. **d** Changes in anti-SjSP-164 antibody. **e** Changes in anti-SjSP-189 antibody. **f** Changes in anti-SjSP-216 antibody

Sera from three infected mice were collected before infection (0 week) and at week 1 to 6 weeks post-infection. Three uninfected mice were used as negative controls. We found that the levels of specific antibodies against all six antigens increased during the course of infection in mouse but that the seroconversion times differed for each antigen (Fig. 2). We detected a significant increase in antibodies against SjSP-13 and SjSP-216 3 weeks post-infection (Fig. 2a and f). This was 1 week earlier than SjSP-160 (Fig. 2c), SjSP-164 (Fig. 2d), and SjSP-189 (Fig. 2e) and 3 weeks earlier than SjSP-23 (Fig. 2b). These results indicated that SjSP-13 and SjSP-216 are candidates for early serological diagnosis in mice.

### Kinetics of specific antibodies in the sera of rabbits infected with *S. japonicum*

We observed a significant increase in antibodies against SjSP-13, SjSP-23, SjSP-160 and SjSP-216 in the three infected rabbits 6 weeks post-infection (Fig. 3). There was no increase in antibodies against SjSP-164 and SjSP-189 during the course of infection (Fig. 3). As in the mouse host, antibodies against SjSP-216 increased significantly just 3 weeks after infection (Fig. 3f). Significant increases in antibodies against SjSP-13 did not occur until 5 weeks post-infection (Fig. 3a). Interestingly, antibodies against SjSP-23 could only be detected 6 weeks post-infection in both the mouse and the rabbit model. Hence, these results indicated only SjSP-216 could serve as a candidate for early diagnosis of schistosomiasis in rabbits.

### Sensitivity and specificity of SjSP-216 as a candidate for early serological diagnosis of *S. japonicum* infections

**Fig. 4:**
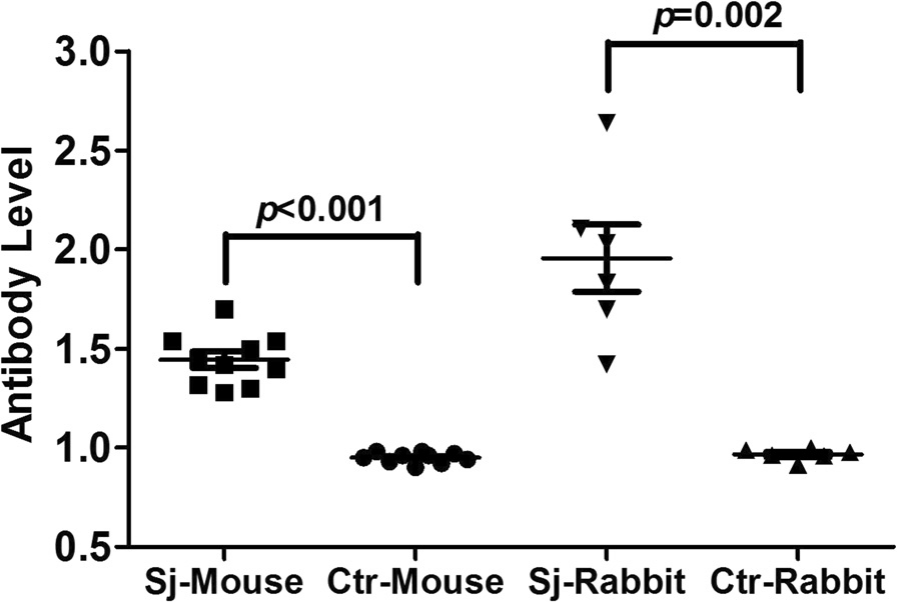
The sensitivity and specificity of SjSP-216-based ELISA for early diagnosis of schistosomiasis. Ten mice and six rabbits were infected with *S. japonicum*. Sera were collected three weeks post-infection. The anti-SjSP-216 antibody levels of each sample were determined from the ratio of the OD_450nm_ values to the cut-off values (determined from the mean plus three standard deviations from the sera, prior to infection). Data were expressed as the mean ± standard error. Sj-Mouse: *S. japonicum* infected mouse. Ctr-Mouse: Uninfected control mouse. Sj-Rabbit: *S. japonicum* infected rabbit. Ctr-Rabbit: Uninfected control rabbit

The results from the above experiments indicated that the SjSP-216 protein had the potential to be a protein marker for early schistosome infection. To further investigate its diagnostic validity of early infection, we carried out additional experiments in the murine and rabbit models of human schistosmiasis with a large sample size (ten mice and six rabbits for both infection group and non-infection group). As shown in Fig. 4, in the mouse experiment, all the serum samples collected from the infected mice at 3 weeks after infection yielded a seropositive reaction (100 % seroconversion) whereas the samples from the uninfected mice showed no seroreaction. Similar to the results in mice, all the serum samples from the infected rabbits at three weeks after infection generated seropositive reactions (100 % seroconversion) and no SjSP-216 specific antibody was detectable in the uninfected rabbits.

## Discussion

Schistosomiasis remains a serious public health problem in developing countries. Morbidity and mortality from this disease are associated with the chronic infection stage, which occurs after egg deposition. Thus, an ideal diagnostic test for schistosomiasis should be capable of detecting parasites as early as possible after the onset of infection. An early diagnostic test would enable more rapid treatment and would interrupt the transmission cycle of the parasite and the progress of the disease. However, the current diagnostic standards for schistosomiasis all depend on the detection of eggs. In addition, as disease is asymptomatic in its early stages, clinical examinations can’t confirm infection. Recently, molecular techniques to detect schistosome infections have been developed to facilitate early diagnosis, but these are expensive and suffer from sampling limitations [22, 23]. Serologic assays to detect antibodies against schistosome antigens, however, have proven useful in the clinical diagnosis of schistosomiasis [24–26].

In definitive hosts, the immune systems must confront four life-cycle stages of the parasite: penetrating cercariae, schistosomula (young worms), adult worms and the eggs that are produced by adult worm pairs [8]. As the host is exposed (internally) to cercariae and schistosomula earlier than they are exposed to eggs, antigens that are highly expressed or solely expressed in one of these two stages are likely be potential candidates for early diagnosis. For example, previous studies of the schistosomula (mechanically transformed cercariae) tegument antigen revealed this antigen had diagnostic value for early infections [19]. In this study, we found that antigens SjSP-13, SjSP-160 and SjSP-216 were highly expressed in young worms, while antigens, SjSP-23, SjSP-164 and SjSP-189 were highly expressed in eggs. These distinct expression profiles correlate with the timing of host immune responses to specific stages. For example, seroconversion of young worm antigen SjSP-216 occurred 3 weeks post-infection, 3 weeks earlier than to the egg antigen SjSP-23.

The immunogenicity of antigens varied between the two host species. Previously, we found that SjSP-164 and SjSP-189 were recognized by the antibodies of human patients [20]. In this study, we report that SjSP-164 and SjSP-189 stimulated humoral immune responses in infected mice but not in rabbits. We found that SjSP-13, which has been verified as a diagnostic marker for human schistosomiasis [20], is also recognized by IgG antibodies in both infected mice and rabbits. Nevertheless, the titers of antibodies in mice and rabbits were much lower than those reported for humans, indicating that the antigenicity of SjSP-13 was weaker in these species than in humans. Importantly, the antigenicity of SjSP-216 in mice and rabbits appeared very similar to that reported for this antigen in humans.

For these reasons, we selected the antigen, SjSP-216, as a candidate for early diagnosis of schistosomiasis. Our results confirmed that SjSP-216 was able to diagnose early schistosome infections in both mice and rabbits with 100 % sensitivity and specificity. Moreover, we observed that anti-SjSP-216 antibody levels increased throughout the period of the parasite exposure. Thus, SjSP-216 would also be useful for diagnosing chronic infections. Although early diagnosis may not be necessary in areas with high endemicity where most patients harbor chronic infections [27], it is vital for detecting new infections, and for ensuring effective disease surveillance in areas where the schistosomiasis has be controlled. In areas where schistosomiasis has been controlled, large-scale disease surveillance at the start of the transmission season could identify (and remove) sources of infection and prevent the recurrence of schistosomiasis. Moreover, early diagnosis will lead to earlier treatment, potentially preventing the development of severe pathologic lesions. This would be particularly relevant for susceptible individuals, such as travellers [28]. Although Praziquantel^TM^ is more effective on adult worms than young worms, high-dose (500 mg/kg) Praziquantel^TM^ therapy or prolonging the course of treatment can damage the tegument or even kill young worms [29, 30].

We propose that SjSP-216 be further developed as an early serological diagnostic tool for human schistosomiasis, as this protein has strong antigenicity in different hosts, including humans. It is difficult to validate the early diagnostic efficacy of SjSP-216 in humans directly as current techniques can’t detect pre-patent human infections. In addition, it is not easy to regularly monitor antibody changes during the course of infection in human. However, it is possible to compare the positive rates of SjSP-216 and other antigens including SjSP-13 at the start of transmission season (i.e. in May) and at the end of epidemic season (i.e. in September). If the sensitivity of SjSP-216 was higher during the epidemic season, we can prove the early diagnostic efficacy of SjSP-216 indirectly.

## Conclusions

This study strongly suggests that SjSP-216, a highly expressed gene in the young worm stage, could serve as a potential biomarker for the early immunodiagnosis of *S. japonicum* infections in vertebrate hosts.

## Additional files

Additional file 1:
**Primers used for gene cloning.** (PDF 29 kb) Additional file 2:
**SDS-PAGE analysis of purified recombinant proteins.** Proteins were expressed in *E. coli* and purified using the Ni-NTA agarose affinity system under denaturing conditions. The purified antigens were re-natured in refolding buffer C7 before SDS-PAGE analysis. (PDF 117 kb) Additional file 3:
**Expression profile of superoxide dismutase (SOD) gene at different developmental stages of** ***S. japonicum.*** Real-time PCR was performed to detect the transcripts of SOD at life stages of cercaria, young worm (Young), adult worm (Adult) and egg. Data were analyzed according to 2 − Ct method using GAPDH as the internal control for each sample. The-fold changes of gene transcriptional level in young worm, adult worm and egg were calculated as compared with cercaria. (PDF 73 kb)
