# Supplementary material for: Evaluation of six novel antigens as potential biomarkers for the early immunodiagnosis of schistosomiasis
Source: Parasit Vectors. 2015 Sep 4;8:447. doi: 10.1186/s13071-015-1048-2 (PMC4558877; doi:10.1186/s13071-015-1048-2)
Supplement: Additional file 1: — Primers used for gene cloning. (PDF 29 kb) [file 13071_2015_1048_MOESM1_ESM.pdf]

Additional file 1: Primers used for gene cloning

Primer	Primer sequence (5'-3')
**SjSP-13 Forward**	CG GGATCC CTTGAAAATTCTGTGTCAC
**SjSP-13 Reverse**	CG CTCGAG CTATTA GTGATGGTGATGGTGATG AATAGTGAATTGAACTAGAAAC
**SjSP-23 Forward**	CG GGATCC GCCAAACAATTATCCAACG
**SjSP-23 Reverse**	CG CTCGAG CTATTA GTGATGGTGATGGTGATG TCATTTATCTTTCAGGCAGTAA
**SjSP-160 Forward**	CG GGATCC ATGAACTATTTTGTACTAAC
**SjSP-160 Reverse**	CG CTCGAG CTATTA GTGATGGTGATGGTGATG TTATGCAAAAGGATATTTC
**SjSP-164 Forward**	CG GGATCC ATGTTTCTAAGTGTATTTTTTATC
**SjSP-164 Reverse**	CG CTCGAG CTATTA GTGATGGTGATGGTGATG TTATACCAATGTTATAATACTG
**SjSP-189 Forward**	CG GGATCC ATGTTTCTAAGTGTATTTCT
**SjSP-189 Reverse**	CG CTCGAG CTATTA GTGATGGTGATGGTGATG TTATACCAATGTTATAATAC
**SjSP-216 Forward**	CG GGATCC ATGTTGAAAATCGCAGTTTG
**SjSP-216 Reverse**	CG CTCGAG CTATTA GTGATGGTGATGGTGATG TTAGGTATTTATAAGTCCAGC
