# Supplementary material for: Evaluation of six novel antigens as potential biomarkers for the early immunodiagnosis of schistosomiasis
Source: Parasit Vectors. 2015 Sep 4;8:447. doi: 10.1186/s13071-015-1048-2 (PMC4558877; doi:10.1186/s13071-015-1048-2)

Additional file 2: SDS-PAGE analysis of purified recombinant proteins. Proteins were expressed in *E. coli* and purified using the Ni-NTA agarose affinity system under denaturing conditions. The purified antigens were re-natured in refolding buffer C7 before SDS-PAGE analysis.

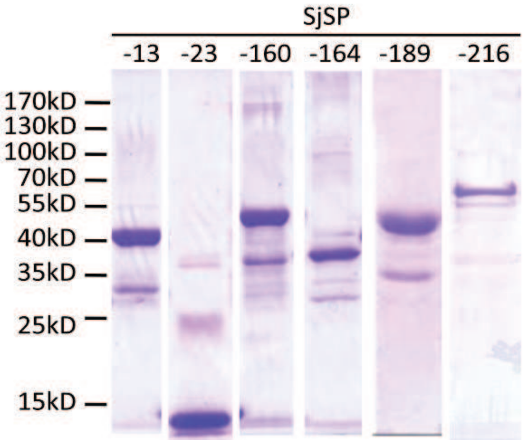

Supplement: Additional file 2: — SDS-PAGE analysis of purified recombinant proteins. Proteins were expressed in E. coli and purified using the Ni-NTA agarose affinity system under denaturing conditions. The purified antigens were re-natured in refolding buffer C7 before SDS-PAGE analysis. (PDF 117 kb) [file 13071_2015_1048_MOESM2_ESM.pdf]
