# Supplementary material for: Evaluation of six novel antigens as potential biomarkers for the early immunodiagnosis of schistosomiasis
Source: Parasit Vectors. 2015 Sep 4;8:447. doi: 10.1186/s13071-015-1048-2 (PMC4558877; doi:10.1186/s13071-015-1048-2)

Additional file 3: Expression profile of superoxide dismutase (SOD) gene at different developmental stages of *S. japonicum*. Real-time PCR was performed to detect the transcripts of SOD at life stages of cercaria, young worm (Young), adult worm (Adult) and egg. Data were analyzed according to $2^{-\Delta\Delta Ct}$ method using GAPDH as the internal control for each sample. The-fold changes of gene transcriptional level in young worm, adult worm and egg were calculated as compared with cercaria.

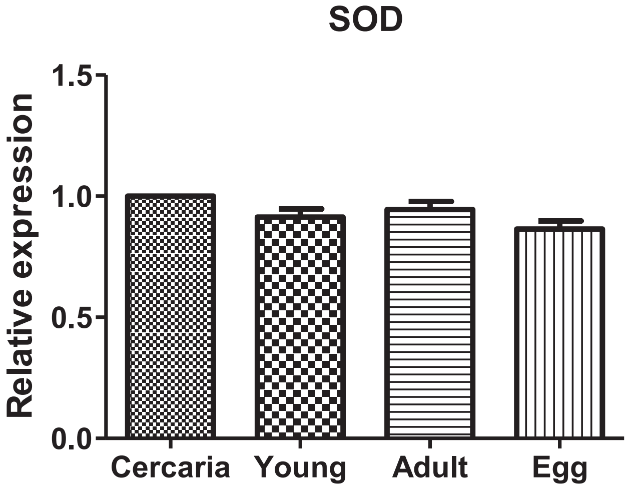

Supplement: Additional file 3: — Expression profile of superoxide dismutase (SOD) gene at different developmental stages of S. japonicum. Real-time PCR was performed to detect the transcripts of SOD at life stages of cercaria, young worm (Young), adult worm (Adult) and egg. Data were analyzed according to 2 − Ct method using GAPDH as the internal control for each sample. The-fold changes of gene transcriptional level in young worm, adult worm and egg were calculated as compared with cercaria. (PDF 73 kb) [file 13071_2015_1048_MOESM3_ESM.pdf]
